# Evaluation of the Potency of the First Commercial Vaccine for *Clostridioides difficile* Infection in Piglets and Comparison with the Humoral Response in Rabbits

**DOI:** 10.3390/vaccines13050438

**Published:** 2025-04-22

**Authors:** Victor Santos do Amarante, João Victor Ferreira Campos, Thayanne Gabryelle Viana de Souza, Yasmin Gonçalves de Castro, Kelly Mara Gomes Godoy, Rodrigo Otávio Silveira Silva

**Affiliations:** Escola de Veterinária, Universidade Federal de Minas Gerais, Avenida Antônio Carlos, 6627, Belo Horizonte 31270-901, MG, Brazil

**Keywords:** diarrhea, CDI, colitis, swine

## Abstract

*Clostridioides difficile* is an anaerobic bacterium that causes disease in both animals and humans. Despite the known significance of this agent, there are no commercial vaccines available for humans, and only one immunogen is marketed for swine. However, no studies have evaluated this vaccine. Background/Objectives: Therefore, the aim of this study was to assess the potency of the first commercial vaccine for *C. difficile* infection in piglets and to compare the humoral response in rabbits and sows. Methods: Pregnant sows were divided into two groups: a vaccinated group (n = 12), receiving two doses before farrowing, according to the manufacturer’s recommendation, and an unvaccinated control group (n = 6). Blood samples were taken from sows and also from piglets up to two days after birth. In addition, two groups of New Zealand rabbits (*Oryctolagus cuniculus*) received either a half-dose (G1) or a full-dose (G2) of the vaccine, with a control group receiving sterile saline (0.85%). Rabbits were vaccinated twice, 21 days apart, with blood samples collected before each dose and 14 days after the final dose. A serum neutralization assay in Vero cells was performed to evaluate the titers of neutralizing antibodies. Results: The vaccine demonstrated immunogenicity by stimulating the production of neutralizing antibodies in both rabbits and sows. Additionally, these antibodies were passively transferred to piglets through colostrum, reaching levels comparable to those found in sows. Furthermore, vaccinated rabbits developed antibody titers that do not significantly differ from those obtained in sows and piglets. Conclusions: The tested vaccine can induce a humoral immune response against *C. difficile* A/B toxins in sows and these antibodies are passively transferred to neonatal piglets through colostrum. Also, the vaccination of rabbits might be a useful alternative for evaluating the potency of vaccines against *C. difficile*.

## 1. Introduction

*Clostridioides difficile* is an obligate anaerobic gram-positive bacillus capable of colo-nizing the intestines of domestic and wild animals, as well as humans [1,2,3,4]. The bacte-rium was first isolated from the feces of healthy human neonates in 1935 [5] and from various domestic animals in the 1980s [6]. Since then, it has undergone several taxonomic reclassifications, having been previously referred to as *Bacillus difficile*, *Clostridium difficile*, *Peptoclostridium difficile*, and, more recently, *Clostridioides difficile* [3,5].

Its ability to form spores allows it to persist in various environments for extended periods, including water, soil, food, and hospital settings [3,7]. In recent decades, *C. difficile* has been identified as one of the leading causes of diarrhea in hospitalized humans, with high morbidity, mortality rates, prolonged hospital stays, and increased treatment costs [3,8]. The use of antimicrobials has been recognized as the primary risk factor for *C. difficile* infection in humans, as it causes dysbiosis that favors colonization and proliferation of this pathogen [7,9].

In the 2000s, the emergence of epidemic strains (formerly referred to as hypervirulent) has contributed to the increased burden and mortality of healthcare-associated *C. difficile* infections [10]. These strains, commonly classified as ribotype 027, exhibit greater pathogenicity and antimicrobial resistance, complicating the elimination of the pathogen, thus enabling prolonged colonization and increasing the recurrence of the disease [10,11,12]. On the other hand, an increased prevalence of the community-acquired *C. difficile* infection has been noted in several countries in the last years [13]. Some of these cases are associated with ribotype 78, commonly found in farm animals worldwide, particularly in pigs [9,14,15,16,17], suggesting that multiple sources may be linked to the infection, including contact with animals and animal-derived products [13,18,19,20]. In fact, there is a high similarity between isolates of human and swine origin, suggesting a possible zoonotic transmission of the agent, reinforcing the need for a One Health approach to this disease [13,19].

In pigs, *C. difficile* infection is common during the first week of life, representing one of the main causes of diarrhea in neonatal piglets in numerous countries, with reports indicating infection rates of up to 92% in farms across Asia and Europe [21,22,23]. The disease affects litters from gilts and sows and affected piglets can develop pasty-to-watery diarrhea, but some animals can be constipated or obstipated. At necropsy, edema of the mesocolon and colitis are the most common lesions seen [24,25]. In this species, economic losses are associated with poor body development, which leads to reduced weight gain in the affected herd [2]. Although uncommon, outbreaks can also occur [25].

Despite the significance of *C. difficile* infection for both animals and humans, there are few strategies to prevent the disease. The use of probiotics has been the focus of several studies aimed at preventing and/or treating *C. difficile* infection; however, there is no consensus on the true effects of their use in animals [26,27,28]. Studies suggest that the administration of non-toxigenic strains in animal species reduces the presence of toxigenic strains in feces and prevents the production of toxins A/B [29,30,31]. However, this product is not commercially available. Therefore, in the last decade, the focus has been on vaccines. Studies have demonstrated that the humoral response plays an important role in the occurrence of *C. difficile* infection in both humans and pigs. Individuals with circulating antibodies against toxins A and B can exhibit lower recurrence rates, milder symptoms, or even an absence of clinical signs [32,33,34]. Thus, the use of immunoprophylactic methods has emerged as an important strategy for preventing and controlling the disease [33,35]. However, to date, there are no commercial vaccines for humans, as several candidate immunogens have been abandoned during advanced stages of research [32,36,37].

In animals, the first and only commercial vaccine was introduced to the market in 2022. Composed of toxoid A and B from *C. difficile* and alpha toxoid from *Clostridium perfringens* type A, the vaccine is intended exclusively for the immunization of sows, which would passively transfer neutralizing antibodies against toxins A and B to their piglets via colostrum. However, there are no published studies assessing the potency of this product in the target species or in laboratory animals. So far, the only available publication is a symposium proceeding suggesting that the vaccine may reduce the occurrence of diarrhea in piglets, thereby also reducing the use of antibiotics [38]. From this publication, it remains unclear which component of the vaccine is responsible for the observed effect. More importantly, no study has evaluated whether A/B toxoids induce a strong humoral response in vaccinated sows or whether the resulting neutralizing antibodies are effectively transferred to piglets via colostrum.

Compared to laboratory animals, testing immunogens in production animals, such as pigs, cattle, sheep, and goats, is more difficult and expensive. Therefore, studies have been conducted to determine which laboratory animals are suitable models for evaluating clostridial vaccines [39]. Currently, the development and quality assessment of clostridial vaccines rely on the vaccination of laboratory animals, typically lagomorphs or rodents [40,41]. On the other hand, studies on *C. difficile* vaccines commonly rely on the hamster model, which is based on the induction of fatal *C. difficile* infection. This model has some marked disadvantages: more restricted access to the species compared to mice, rats, and rabbits and ethical concerns due to the severe suffering caused by infection [42,43,44,45]. However, no studies have compared the potency of a *C. difficile* toxoid between rabbits and sows, which could help determine whether lagomorphs serve as a suitable initial model for developing and evaluating vaccines targeting swine. Therefore, the aim of this study was to assess the potency of the first commercial vaccine for *C. difficile* infection in piglets and to compare the humoral response in rabbits and sows.

## 2. Materials and Methods

### 2.1. Experimental Design

So far, there are no official parameters for evaluating the potency of vaccines against *C. difficile* infection. Thus, potency assessment in rabbits (*Oryctolagus cuniculus*) used in the present study was based on the guidelines outlined in the Code of Federal Regulations 9 (CFR9) for *Clostridium sordellii* and *C. perfringens* [40].

### 2.2. Vaccination of Rabbits

**Figure 1:**
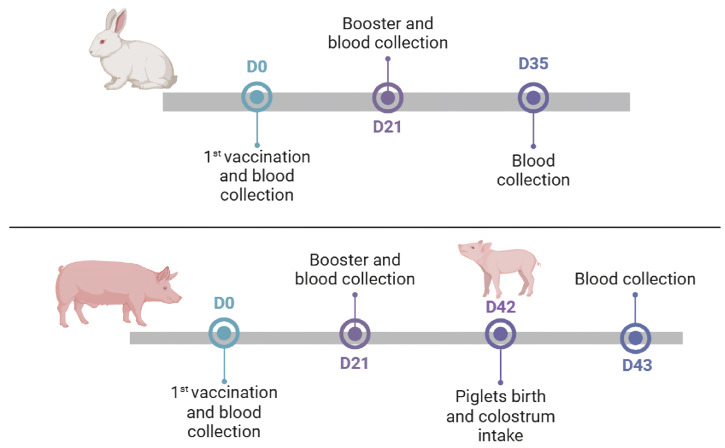
Vaccination and blood collection scheme for experimental groups, consisting of rabbits and pigs.

Two experimental groups of New Zealand rabbits (*Oryctolagus cuniculus*) weighing between 1.5 and 2 kg were employed. Group 1 (G1) was vaccinated with half (1 mL) of the recommended dose of the commercial vaccine (n = 8); Group 2 (G2) received the full dose of the commercial vaccine (2 mL, n = 8). A control group (C1) was also included, in which animals received 2 mL of 0.85% sterile saline solution (n = 6). All rabbits received two doses spaced 21 days apart, and serum samples were collected before each vaccination and 14 days after the final dose (Figure 1). This study was approved by the Ethics Committee on Animal Use (Comissão de Ética no Uso de Animais da Universidade Federal de Minas Gerais—CEUA-UFMG, protocol 277/2020).

### 2.3. Vaccination of Sows

A commercial farm in Minas Gerais, Brazil, with 600 sows, was selected to evaluate the vaccine. Around 15 days before the study began, fecal samples were randomly collected from 10 piglets from different litters and 30 sows. These specimens were subjected to A/B toxins detection (Ridascreen *C. difficile* toxins A/B, R-Biopharm, Darmstadt, Germany) and the isolate *C. difficile*, as previously described [10]. Briefly, equal volumes of stool samples and 96% ethanol (*v*/*v*) were mixed and incubated for 30 min at room temperature. Thereafter, 20 µL aliquots were inoculated on plates containing cycloserine-cefoxitin-fructose agar, supplemented with 7% horse blood and 0.1% sodium taurocholate (Sigma-Aldrich Co., Saint Louis, MO, USA). Following anaerobic incubation at 37 °C for 72 h, all colonies with suggestive morphology (flat, irregular, and with ground-glass appearance) were subjected to a previously described multiplex-PCR for a housekeeping gene (*tpi*), the toxin A gene (*tcdA*), the toxin B gene (*tcdB*), and a binary toxin gene (*cdtB*) [10,46].

All animals tested negative for A/B toxins and also for the presence of *C. difficile*. Two experimental groups of pregnant sows were used: the vaccinated group (n = 12) received two doses of the commercial vaccine at six and three weeks before birth (T0 and T1, respectively), as recommended by the manufacturer. Simultaneously, the animals also received vaccinations against other agents that cause neonatal diarrhea, as per the farm’s routine (SUISENG Coli/C, HIPRA, Amer, Spain). The control group (n = 6) was maintained according to the farm’s routine and was not immunized with the *C. difficile* toxoid. Before each vaccination, 5 mL of blood was collected from each sow via jugular puncture [47].

### 2.4. Passive Immunity Assessment

Piglets were subjected to assisted colostrum intake, similar to previous studies [48,49]. Each piglet was monitored to ensure it successfully reached a teat and consumed colostrum immediately after birth. Once a piglet finished suckling, it was marked and separated to allow the remaining neonates uninterrupted access to colostrum, ensuring all animals ingested similar amounts. Between 24 and 48 h after birth, blood samples were collected from the sows (T2), and six piglets from each sow were randomly selected for blood collection via jugular vein puncture [47]. This study was approved by the Ethics Committee on Animal Use (Comissão de Ética no Uso de Animais da Universidade Federal de Minas Gerais—CEUA-UFMG, protocol 184/2022).

### 2.5. Sera Titration

Neutralizing antibody titration was conducted using serum neutralization in cells, similar to previous studies testing other clostridial vaccines [39,50]. To perform the serum neutralization test, the native A/B toxins were produced via the dialysis method as previously described [51,52]. Briefly, a fully characterized isolate (EQ5) from our collection was used. This strain was originally isolated in a previous study [53] and it was selected because it belongs to ribotype 078 and sequence type 11 (clade 5), which is commonly associated with *C. difficile* infection in piglets worldwide.

For A/B toxin production, EQ5 was first cultured on Brain Heart Infusion broth (BHI, Oxoid, UK) under anaerobic conditions (Anaerobic Chamber Model 1025, Thermo Fisher Scientific, Waltham, MA, USA) for 36–28 h. Subsequently, 100 µL of the culture was inoculated in a 14 kDa dialysis sack (Sigma-Aldrich Co., Saint Louis, MO, USA) filled with phosphate-buffered saline (PBS) as previously described [51,52]. The dialysis sack was then immersed in a flask containing 500 mL of BHI broth (Oxoid, UK) supplemented with 0.1% L-cysteine. The apparatus was incubated at 37 °C in aerobic conditions for 72 h. Following incubation, the contents of the dialysis sack were centrifuged at 10,000× *g* for 30 min at 4 °C and filtered through a 0.22 µm membrane. The resulting toxin preparation was then stored at −80 °C until use.

Using the standard *C. sordellii* antitoxin as a reference (NIBISC, England) [54], the A/B toxin was standardized at the level of test L+/150 (or 0.06 IU/mL) in African green monkey kidney cells (VERO cells, ATCC CCL-81) at a concentration of 5 × 10^4^ per well [55]. The title was standardized at the highest dilution where cell rounding was observed in >90% of the cells [56]. Back-titration using the standard antitoxin at 10 IU/mL, 5 IU/mL, 2 IU/mL, and 1 IU/mL was used to confirm the standardization of the toxin.

The titration of sera from sows and rabbits was performed individually, whereas piglet sera were analyzed in pools of three littermates (two pools per sow, totaling 36 samples) due to limited serum volume. As a quality control, standard *C. sordellii* antitoxin (National Institute for Biological Standards and Control—NIBSC, UK), diluted to 1 IU/mL and 2 IU/mL, was included in all experiments.

### 2.6. Statistical Analysis

The results were analyzed using Prism software 10.4.2 (GraphPad, Boston, MA, USA). Homoscedasticity and normality of data were assessed using the F-test and Shapiro–Wilk test, respectively. In the absence of normal data distribution, the Mann–Whitney test was used to verify differences between the control and vaccinated groups, and the Wilcoxon test was used to check differences within the same group over time. The correlation between the response of sows and their piglets was evaluated using the non-parametric Spearman method.

## 3. Results

### 3.1. Immunogenicity in Sows

**Figure 2:**
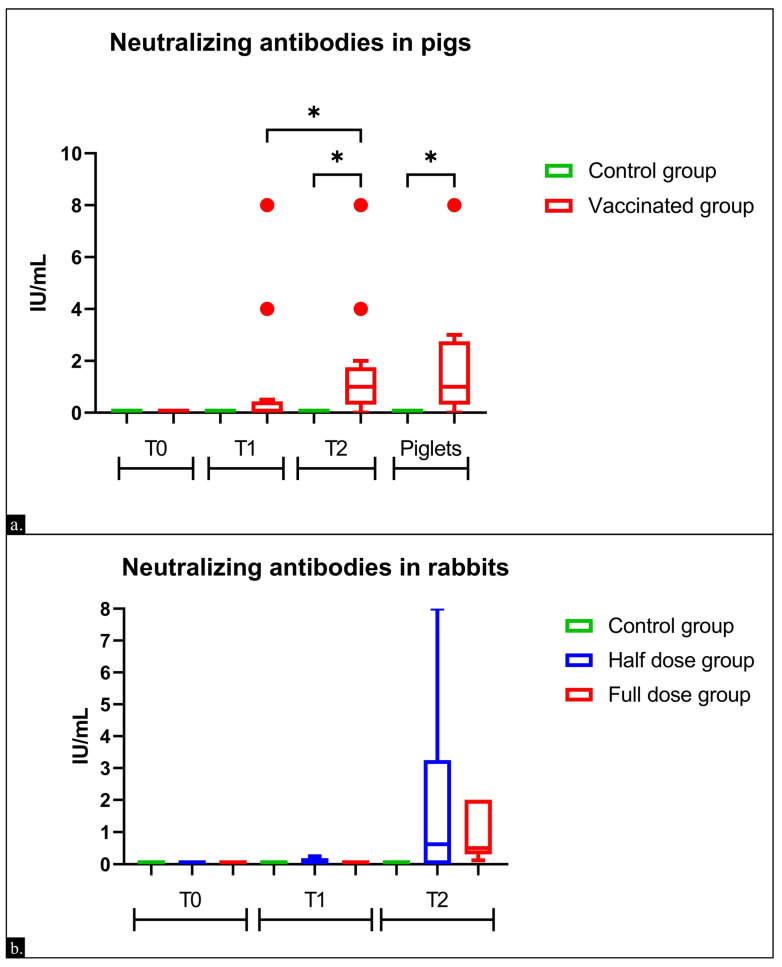
Average titers of neutralizing IgG antibodies to *C. difficile* toxins A/B (**a**) in sows and piglets; (**b**) in rabbits. The test detection level was 0.06 IU/mL. Statistical significance was assessed using Welch’s *t*-test. * *p* < 0.05.

Before the first dose, all sows tested negative for neutralizing antibodies against toxins A/B (Figure 2). Furthermore, sera from sows in the control group and their piglets did not show neutralizing activity at any phase of this study. After the first dose (T1), the sows in the vaccinated group had an average titer of 1.06 ± 2.46 IU/mL, with four animals (33.3%) showing seroconversion. After the second dose (T2), the average antibody titer in the vaccinated group increased to 1.60 ± 2.29 IU/mL, with 10 animals (83.3%) showing seroconversion. Piglets from vaccinated sows had an average titer of 1.74 ± 2.22 IU/mL. A correlation of 91.52% was observed between the neutralizing antibody titers of the immunized sows and their respective piglets (*p* < 0.001).

### 3.2. Immunogenicity in Rabbits

Before the first dose, all rabbits tested negative for neutralizing antibodies against toxins A/B. At three weeks after the first dose (T1), two animals (25%) in the group vaccinated with half the dose (G1) exhibited neutralizing antibodies, with an average titer of 0.06 ± 0.11 IU/mL, whereas the group that received the full dose showed no detectable humoral response (Figure 2). After the second dose (T2), the average antibody titer in the group vaccinated with the full dose (G1) was 0.98 ± 0.85 IU/mL, with all animals showing seroconversion. In the group that received half the dose (G2), the average titer was 1.78 ± 0.11 IU/mL, with five animals (62.5%) exhibiting a humoral response. The control group remained negative for neutralizing antibodies throughout the duration of the experiment.

### 3.3. Comparison of Humoral Responses in Rabbits, Sows, and Piglets

**Figure 3:**
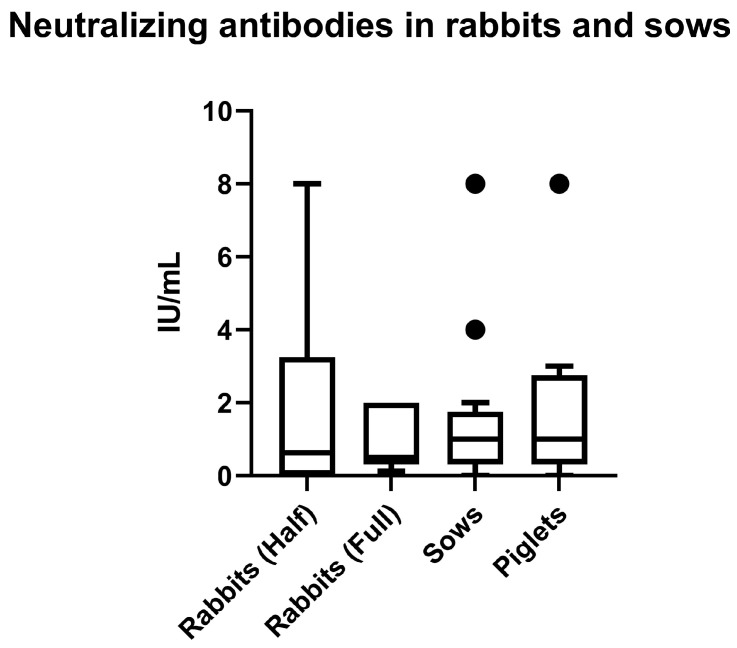
Comparison between groups of vaccinated rabbits and pigs with two doses of the vaccine against *C. difficile* infection.

A comparison between groups of vaccinated rabbits and sows or piglets was performed using the non-parametric Kruskal–Wallis test. There was no statistical difference between the two groups of rabbits and sows vaccinated with two doses of the vaccine (*p* = 0.8566) (Figure 3).

## 4. Discussion

*C. difficile* infection is a significant cause of neonatal diarrhea in pigs, resulting in economic losses primarily associated with impaired body development [35,57]. In some countries, *C. difficile* is recognized as the primary cause of enteric disease in animals aged 1 to 7 days [58,59,60]. In general, piglets are not treated for *C. difficile* infection due to the number of animals that can be affected, increasing the costs of medication and management [61], so the focus is on the prevention of the disease. In addition, the growing presence of this pathogen in pigs remains a public health concern, as animals can serve as reservoirs for toxigenic *C. difficile* strains and also for antimicrobial resistance determinants [62,63,64]. Some studies have also suggested that *C. difficile* infection may be a zoonotic disease [65,66]. Notably, prevention of *C. difficile* infection remains a challenge also in humans, as most vaccine studies to date have been terminated during the clinical analysis phases [36,37,67].

Despite the recognized importance of *C. difficile* infection, until recently, there were no commercial vaccines available to prevent this enteric disease in animals and humans. In 2022, the first the only immunogen to control the disease in pigs was introduced to the market in several countries, including Brazil and several European countries. The present study revealed, for the first time, that this immunogen against *C. difficile* diarrhea is capable of inducing neutralizing antibodies in pregnant sows. The transfer of passive immunity from sows to piglets was also demonstrated. Additionally, this study suggests a high similarity in the immune response between rabbits, sows, and piglets, indicating that rabbits can be used in research on the development and evaluation of *C. difficile* vaccines.

Considering that *C. difficile* infection affects neonatal piglets, the passive transfer of antibodies against A/B toxins is believed to be a possible way to protect newborns [68,69,70]. In the present study, antibody titers were maintained after the second dose of the vaccine, ensuring the presence of circulating IgG in pregnant sows during the peripartum period: after two doses of the vaccine, more than 80% of the vaccinated sows exhibited seroconversion. As expected for a clostridial toxoid, the average neutralizing antibody titers after the second dose were higher compared to those after the first dose (Figure 2). A high concentration of circulating antibodies in sows just before farrowing is known to increase the chance of their mobilization into colostrum, enhancing transfer to piglets [71,72,73]. Further studies should evaluate the duration of this humoral response and the effect of multiple immunizations, as the manufacturer’s recommendations suggest revaccination with one dose per pregnancy in sows [74].

Previous studies with pigs have shown that the passive transfer of human monoclonal antibodies or bovine hyperimmune colostrum can protect the intestinal mucosa of piglets and prevent the systemic action of toxins [75,76]. Since *C. difficile* infection in pigs affects piglets in the first week of life, these findings paved the way for vaccine research focusing on the active immunization of sows with subsequent transfer of neutralizing antibodies via colostrum [68,77]. In this context, the present study revealed a strong correlation between the titers obtained in sows after two doses of the vaccine and the titers observed in piglets immediately after birth, indicating efficient transfer of neutralizing antibodies via colostrum with the evaluated vaccine, similar to previous studies with other immunogens in swine [47,78,79,80]. It is important to note that the piglets in this study underwent assisted colostrum intake, which is not the standard practice on most farms. Therefore, further studies are needed to better understand how different management practices influence the titers of neutralizing antibodies against toxins A and B in piglets.

It is also noteworthy that, beyond the initial absorption of immunoglobulins in the first hours of life, these antibodies continue to be ingested through the ongoing intake of colostrum, transitional milk, and, later, mature milk [81]. Thus, in addition to being absorbed into the circulation, these antibodies may also provide local protection, as immunoglobulins present in the intestinal lumen could help neutralize *C. difficile* toxins, thereby protecting the animals against *C. difficile* infection or reducing the severity of clinical signs [75,76].

Several strategies for the prevention of *C. difficile* infection have been proposed so far. The use of monoclonal IgG targeting toxin B (known as bezlotoxumab) was shown to significantly reduce disease recurrences and is currently used in certain cases in human patients [36,82,83]. The use of probiotics, particularly *Saccharomyces boulardii*, has attracted considerable attention, but it is still not recommended by the main international guideline [84]. Also, the applicability of probiotics in lactating piglets would be limited. The administration of non-toxigenic *C. difficile* strains has also been proposed as a preventive strategy in swine and humans, but no commercial products are currently available [29,30,31]. Consequently, the development of immunogens targeting toxins A and B has become the primary focus of many companies and research groups. In this context, both Pfizer and Sanofi Pasteur discontinued their vaccine candidates against *C. difficile* infection in humans, underscoring the challenges associated with developing effective vaccines for this pathogen [85,86]. More recently, an mRNA-based vaccine has shown promising results in limiting acute disease and promoting bacterial decolonization [86]; however, further studies are needed to confirm its efficacy and practical applicability. One of the main challenges in developing a vaccine strategy against *C. difficile* infection in humans is the impaired immune response observed in vaccinated individuals. In contrast, a key advantage of vaccination in swine is that it is administered to immunocompetent sows, allowing for the passive transfer of immunoglobulins to the target animals—neonatal piglets. Additionally, the continuous ingestion of milk containing these antibodies represents another benefit over the human context, potentially enhancing the effectiveness of the immunogen in this species.

The present study confirms that the tested commercial vaccine can induce a humoral immune response against *C. difficile* A/B toxins in immunized sows and that these antibodies are effectively transferred to neonatal piglets through colostrum. Although this finding is promising, it should be interpreted with caution, as it does not confirm that the vaccine can prevent *C. difficile* infection. The next steps of this study involve evaluating the ability of this immunogen to prevent the disease in hamsters, a widely used animal model for *C. difficile* infection studies [10,43]. Subsequently, a large-scale field study will be conducted to assess how this toxoid impacts the prevalence of the disease in the short, medium, and long term in commercial farms. If its ability to prevent *C. difficile* infection is confirmed, this vaccine would represent a significant advancement not only for pig production but also from a One Health perspective, given the potential zoonotic nature of the agent [13,19].

The development and quality assessment of clostridial vaccines require evaluation in animal models [87,88]. In this context, testing immunogens in production species such as swine is more challenging and costly compared to laboratory animals. As a result, clostridial vaccine development and quality control efforts commonly rely on laboratory animals, typically lagomorphs or guinea pigs [43,89,90]. However, no studies have compared the potency of a *C. difficile* toxoid between rabbits and sows, so it is unknown if this species can be used as a model for the development and quality assessment of *C. difficile* vaccines.

Unfortunately, there are no established parameters to predict the clinical efficacy of vaccines against *C. difficile* infection. Anyway, the present results confirm the immunogenicity of the evaluated vaccine and its ability to induce neutralizing antibodies in a rabbit model, which is commonly employed for the evaluation of several clostridial toxoids [89,91,92]. Notably, no differences were observed in the humoral immune response between rabbits and the target species (sows or piglets), suggesting that the use of rabbits in the development and assessment of *C. difficile* vaccines may be a viable strategy. This finding aligns with previous studies on clostridial toxoids and vaccines for viral diseases, which proposed rabbits as a suitable initial model for developing vaccines targeting swine species [93,94]. In fact, in several countries, the evaluation of clostridial vaccines relies on the systematic vaccination of laboratory animals due to their proven immunological similarity to target domestic species [40,91,92,95] as well as their cost-effective and easy handling [93,96].

Given that the present study evaluated only a single vaccine formulation, further research is necessary to confirm the suitability of the rabbit model for testing *C. difficile* toxoids. If validated, the use of rabbits could also contribute to reducing reliance on the hamster model for *C. difficile* vaccine evaluation. Although the hamster model—based on the induction of fatal *C. difficile* infection—is well characterized, it presents several disadvantages: limited availability of immunological tools, more restricted access to the species compared to mice, rats, and rabbits, and ethical concerns due to the severe suffering caused by the rapid onset of acute colitis following infection [42,43,44,45].

## 5. Conclusions

The present study concludes that the tested commercial vaccine can induce a humoral immune response against *C. difficile* A/B toxins in immunized female swine. Furthermore, these antibodies are passively transferred to neonatal piglets through colostrum. Also, vaccinating rabbits appears to be an effective method for evaluating the potency of vaccines against *C. difficile*, yielding results that do not differ statistically from those obtained in sows and piglets.

## Data Availability

All data presented in this study are available on request from the corresponding author.
